# Management of ovarian and endometrial cancers in women belonging to HNPCC carrier families: review of the literature and results of cancer risk assessment in Polish HNPCC families

**DOI:** 10.1186/s13053-015-0025-2

**Published:** 2015-01-16

**Authors:** Tadeusz Dębniak, Tomasz Gromowski, Rodney J Scott, Jacek Gronwald, Tomasz Huzarski, Tomasz Byrski, Grzegorz Kurzawski, Dagmara Dymerska, Bohdan Górski, Katarzyna Paszkowska-Szczur, Cezary Cybulski, Pablo Serrano-Fernandez, Jan Lubiński

**Affiliations:** 1 Department o f Genetics and Pathology, International Hereditary Cancer Center, Pomeranian Medical University, Szczecin, Poland; 2 Discipline of Medical Genetics, Faculty of Health, University of Newcastle and Hunter Medical Research Institute, Newcastle, NSW Australia

**Keywords:** HNPCC, Lynch syndrome, Ovarian cancer, Endometrial cancer, Adnexectomy

## Abstract

**Background:**

Over half the cancer deaths in HNPCC families are due to extra-colonic malignancies that include endometrial and ovarian cancers. The benefits of surveillance for gynecological cancers are not yet proven and there is no consensus on the optimal surveillance recommendations for women with MMR mutations.

**Methods:**

We performed a systematic review of the literature and evaluated gynecological cancer risk in a series of 631 Polish HNPCC families classified into either Lynch Syndrome (LS, MMR mutations detected) or HNPCC (fulfillment of the Amsterdam or modified Amsterdam criteria).

**Results:**

Published data clearly indicates no benefit for ovarian cancer screening in contrast to risk reducing surgery.

We confirmed a significantly increased risk of OC in Polish LS families (OR = 4,6, p < 0.001) and an especially high risk of OC was found for women under 50 years of age: OR = 32,6, p < 0.0001 (95% CI 12,96-81,87). The cumulative OC risk to 50 year of life was calculated to be 10%. Six out of 19 (32%) early-onset patients from LS families died from OC within 2 years of diagnosis. We confirmed a significantly increased risk of EC (OR = 26, 95% CI 11,36-58,8; p < 0,001). The cumulative risk for EC in Polish LS families was calculated to be 67%.

**Conclusions:**

Due to the increased risk of OC and absence of any benefit from gynecological screening reported in the literature it is recommended that prophylactic oophorectomy for female carriers of MMR mutations after 35 year of age should be considered as a risk reducing option. Annual transvaginal ultrasound supported by CA125 or HE4 marker testing should be performed after prophylactic surgery in these women.

Due to the high risk of EC it is reasonable to offer, after the age of 35 years, annual clinical gynecologic examinations with transvaginal ultrasound supported by routine aspiration sampling of the endometrium for women from either LS or HNPCC families. An alternative option, which could be taken into consideration for women preferring surgical prevention, is risk reducing total hysterectomy (with bilateral salpingo-oophorectomy) for carriers after childbearing is complete.

**Electronic supplementary material:**

The online version of this article (doi:10.1186/s13053-015-0025-2) contains supplementary material, which is available to authorized users.

## Background

Lynch syndrome, also referred to as hereditary non-polyposis colorectal cancer (HNPCC), accounts for somewhere between 2 and 5% of all CRC [1],[2]. It has been shown that Lynch Syndrome (LS) is a result of germline mutations in genes involved in DNA mismatch repair (MMR) MSH2, MLH1, MSH6, and PMS2, whereas as HNPCC refers to families that adhere to the Amsterdam criteria or iterations of it. Mutations within MSH2 and MLH1 are the most frequently observed in Lynch syndrome [3]-[6]. More recently, it has been reported that loss of EPCAM is associated with Lynch syndrome, by virtue of it changing the epigenetic status of the promoter region of MSH2 [7]. Mutation carriers are at high risk of developing colorectal cancer (CRC), and endometrial cancer (EC) at unusually young ages [8]. Other, extra-colonic tumor types such as ovarian, small bowel, urinary, biliary tract, gastric, and brain tumors, have also been associated with HNPCC [9],[10]. Due to the high cumulative risk of CRC colonoscopy is recommended in LS families [11]. This strategy has been documented to decrease CRC mortality [12]. Over half the cancer deaths in HNPCC families are due to extra-colonic malignancies [13]. The benefit of surveillance for gynecological cancers is not yet proven and there is no consensus on the optimal surveillance recommendations for women with MMR mutations. The aim of this study was to perform a systematic review of the literature and to evaluate and compare cancer risk in our series of 631 Polish HNPCC families including 279 families with identified mismatch repair (MMR) gene mutations (referred as Lynch syndrome (LS) families) in order to suggest optimal management of ovarian and endometrial cancer for female MMR mutation carriers and their close relatives.

## Material and methods

The relevant literature articles were found after a comprehensive search of the Medline (Pubmed) database from its inception to 17 September 2014 by inputting “HNPCC”; 4298 publications were identified. A refined search using combinations of terms: “HNPCC”, “tumor spectrum”, “ovarian cancer”, “endometrial cancer”, “screening”, “cancer risk”, “surveillance” and “prophylactics” identified reference used herein.

During 2002-2014 631 HNPCC families studied herein were classified into either LS or HNPCC at the International Hereditary Cancer Center (IHCC) Szczecin, Poland, due to 1): identification of pathogenic MMR gene mutations -278 LS families (1176 women, 1507 men); or 2): fulfillment of Amsterdam criteria (144 families, 240 affected individuals and 750 their first-degree relatives) or HNPCC suspected criteria (at least 2 first degree relatives affected with CRC/EC and at least one of them diagnosed < 50 yrs- 209 families, 348 affected patients and 1092 their first-degree relatives) – total 353 families referred as HNPCC families (with no MMR mutations) (1285 females, 1145 males). There were 1433 mutation carriers and 1250 first-degree relatives in LS families: 1371 individuals coming from 142 MLH1 families, 946 individuals from 98 MSH2 families, 347 persons from 36 MSH6 families, 19 individuals from 2 EPCAM families.

Patients originated from different regions of Poland. All adult LS mutation carriers or first-degree relatives were included, as well as probands and their adult first degree-relatives from HNPCC families. We investigated the tumor spectrum, age of onset of ovarian cancer (OC) and endometrial cancer (EC), the risk of OC and EC and survival from OC in these families. Observed (OF) and expected frequencies (EF) were calculated by the evaluation of the total number of family members and affected individuals in different age groups (range of 5 years) in LS and HNPCC families and compared to age-specific incidence rates in the different age groups (range 5 years) per 100,000 people. These calculations were matched by site with the individuals registered in the Polish general population [14]. Statistical analyses including evaluation of expected and observed frequencies and cumulative risk were performed using Cox regression, chi-square with Yates correction and *T*-test analysis.

All participants signed an informed consent document prior to entering the study. The study was approved by the institutional review board of the Pomeranian Medical University.

For 10-year survival analysis a comparison with follow-up data from a consecutive ovarian cancers was performed. This group consisted of 608 unselected ovarian cancer patients (there were no HNPCC- and LS-associated OC in this series of cases) from the registry of International Hereditary Cancer Centre in Szczecin, Poland, who were diagnosed from 1998 to 2006 in cooperating Oncology Centers in Szczecin, Poznań and Rzeszów. All cases were histologically or cytologically confirmed.

## Results

### Tumor spectrum

#### Literature data

Recent analysis of the cancer spectrum in 368 MMR genes mutation carriers (mainly non-Hispanic white US citizens) from 176 families confirmed that the two most common LS cancers were: CRC (58% of all cancers) in both sexes and EC (14%) followed by ovarian cancer (OC) as the third most common malignancy (3,5%). Cancers of the urogenital tract (kidney/uterus/bladder) constituted 3,1%, stomach/small intestine 2,7%, breast 1,9% and prostate 1.1% of all malignancies in these families [15]. Another study from Europe performed on 2118 German and Dutch MMR gene mutation carriers revealed a similar tumor spectrum and a high incidence of gynecological cancers: CRC 50%, EC 16%, OC 4,4%, breast 4,4%, urological 3,6%, stomach 1,6% [16]. LS-associated OC has been reported to exhibit a variety of histopathological subtypes, mostly invasive, with 22% presenting with synchronous primary EC [17].

#### Polish data

Consistent with reports in the literature, a comparison of the cancer spectrum between 278 LS families and 353 HNPCC families diagnosed at the IHCC confirmed the high incidence of gynecological cancers in Polish HNPCC families. There were 21 OCs among 573 tumors (3,6%) in LS families and 18 OCs among 588 tumors (3,1%) in the HNPCC families. EC was more prevalent among LS families (138/573 tumors, 24% of all cancers) compared to HNPCC families (81/588 tumors, 14% of all cancers). There were no major differences in the distribution of other tumors between either the LS or HNPCC families (Table 1).

**Table 1:** Cancer spectrum in MMR genes mutation positive and negative HNPCC families

		Mutation positive						Mutation negative			
	N	Mean age	%	<50	≥50		N	Mean age	%	<50	≥50
CRC	289	48,2	50,4 [289/573]	171	118	CRC	345	53,4	58,6 [345/588]	156	189
EC	138	49,8	24,1 [138/573]	69	69	EC	81	51	13,7 [81/588]	41	40
OC	21	42,6	3,66 [21/573]	19	2	OC	18	54,6	3,06 [18/588]	5	13
KI	6	52,2	1,04 [6/573]	3	3	KI	13	57,3	2,21 [13/588]	3	10
BL	5	55,4	0,87 [5/573]	3	2	BL	6	49	1,02 [6/588]	3	3
LC	3	60	0,52 [3/573]	1	2	LC	2	68,5	0,34 [2/588]	0	2
ST	12	52,6	2,09 [12/573]	6	6	ST	9	49,2	1,53 [9/588]	6	3
PC	6	55,2	1,04 [6/573]	2	4	PC	5	56,4	0,85 [5/588]	1	4
Liv	8	47,6	1,39 [8/573]	5	3	Liv	4	60	0,68 [4/588]	1	3
FGT	36	50,7	6,28 [36/573]	17	19	FGT	32	51,4	5,44 [32/588]	14	18
BR	28	52	4,88 [28/573]	9	19	BR	32	51	5,44 [32/588]	16	16
BONES	4	59,2	0,70 [4/573]	1	3	Thr	1	51	0,17 [1/588]	0	1
Gallbladder	1	50	0,17 [1/573]	0	1	SS	1	58	0,17 [1/588]	0	1
CSU	9	51,4	1,57 [9/573]	4	5	CSU	25	52,3	4,25 [25/588]	10	15
SKIN	2	48,5	0,35 [2/573]	1	1	SKIN	2	59,5	0,34 [2/588]	1	1
MM	1	44	0,17 [1/573]	1	0	MM	1	72	0,17 [1/588]	0	1
Lx	1	52	0,17 [1/573]	0	1	OUN	2	35,5	0,34 [2/588]	1	1
OUN	3	42	0,52 [3/573]	2	1	LEUC	6	50,7	1,02 [6/588]	2	4
LEUC	2	63	0,35 [2/573]	0	2	Salivary gland	1	33	0,17 [1/588]	1	0
LYMPH	1	60	0,17 [1/573]	0	1						

SS-soft tissue sarcoma, CRC-colorectal cancer, EC-endometrial cancer, BL-bladder cancer, KI- kidney cancer, OC- ovarian cancer, LC- lung cancer, ST- stomach cancer, PC- prostate cancer, Liv- liver cancer, FGT- female genital tract, BR- breast cancer, Thr- thyroid cancer, CSU- cancer suspected unknow, MM- malignant melanoma, Lx- larynx cancer, OUN- central nervous system cancer, LEUC- leukemia, LYMPH- lymphoma.

In the LS families individual at risk for CRC was 10,7% (289 CRC among 2683 individuals), for EC 12% (138 cases among 1176 females), for OC 1,7% (21 patients among 1176 women) and 2,8% for early-onset cases (19 cases among 661 women before 50). In the HNPCC families individual at risk for CRC was 14,1% (345 CRC among 2430 individuals), for EC 6,3% (81 cases among 1285 females) and 1,4% for OC (18 patients among 1285 women).

### Risk of ovarian cancer

#### Literature data

For female LS patients, the lifetime risk of developing OC is estimated to be somewhere between 3% and 20% with a standardized incidence ratio (SIR) ranging from 7 to 14 [18]-[22]. The mean age of OC has been reported to be between 40 and 47 years of age [16],[21],[22]. In a recent Danish study the mean age of OC was reported to be lower in LS families (41 years) compared to HNPCC families (66 years) or HNPCC-suspected families (64 years) [23].

#### Polish data

Statistical analysis of the age of onset of gynecological cancers in Polish LS patients reported herein confirmed that the mean age of OC was significantly lower in these families (43 years, age range 31-52) when compared to the general Polish population (54 years, p < 0.0001) and to HNPCC families (53 years, age range 27-80, p < 0.001).

Statistical analyses of the observed (OF = 21 cases) and expected frequency (EF = 4,6) of OC in our series of LS families showed a significantly increased risk of OC (OR = 4,6, 95% CI 2,75-7,78; p < 0.001) in comparison to the general population estimates. An especially high risk of OC was found for women under 50 years of age: OR = 32,6, 95% CI 12,96-81,87; p < 0.0001 (OF = 19 and EF = 0,6). The cumulative OC risk to 50 year of age was calculated to be 10% for Polish female LS patients.

Statistical comparison of the OF (n = 18) and EF (n = 5,4) of OC in our series of HNPCC families showed also significantly, although to a lesser degree than in LS families, increased risk of OC (OR = 3,6, 95%CI 1.34-9.82; p = 0.012). The cumulative OC risk to 85 year of life was calculated to be 6% for Polish females from HNPCC families.

Out of 21 patients affected by OC in the LS families 11 patients harboured MSH2 mutations, 8 females were MLH1 mutations carriers and 2 carried MSH6 mutations.

### Risk of endometrial cancer

#### Literature data

For female LS patients, the lifetime risk of developing EC is estimated to be between 30% and 70% with a standardized incidence ratio (SIRs) ranging from 10 to 62 [21],[22],[24],[25]. The mean age of diagnosis for EC has been reported to be 48, 49 and 54 years in MLH1, MSH2 and MSH6 mutation carriers respectively [26],[27].

#### Polish data

The mean age of EC diagnosed in our LS families was 50 years of age (range 27-78). The mean age of EC detected in HNPCC families was 51 years (range 20-75). Statistical analyses of the observed (OF = 138 cases) and expected frequency (EF = 5,5 cases) of EC in our series of LS families showed a significantly increased risk of EC when compared to general population (OR = 26, 95% CI 11,36-58,8; p < 0,001). The cumulative risk for EC in Polish LS families was calculated to be 67%. Similarly, although to a lesser extent than in LS families, statistical analyses of the OF (n = 81) and EF (n = 6,5) of EC in our HNPCC families revealed a significantly increased risk of EC (OR = 14, 95%CI 6,23-32,98; p < 0,0001). The cumulative risk for EC in Polish HNPCC families was calculated to be 36%.

### Cancer deaths

#### Literature data

In a recent study of 179 Finnish LS families from 1069 mutation carriers 151 had succumbed - 97 (64%) to cancer. From these 55,3% cancer deaths were due to extra-colonic, extra-endometrial cancers; CRC accounted for 36,4% of the deaths; 8.2% due to EC. Only 7.9% of the patients with CRC had died from CRC and 5% of those with EC, respectively; 61% of the extra-colonic, extra-endometrial cancer patients died from their primary disease [13].

Conflicting data regarding prognosis of OC in the HNPCC exists in the literature. A comparison of 26 OC from Dutch HNPCC families versus 52 sporadic OC matched for age, stage and year of diagnosis, derived from the Dutch population revealed that the survival rate was not significantly different between patients with OC-HNPCC and the controls with sporadic OC [28]. There is, however, a second report suggesting that a better prognosis of OC in LS, with a 10-year survival of 81%, than in BRCA1/2 mutation carriers or in the general population [29].

#### Polish data

**Figure 1:**
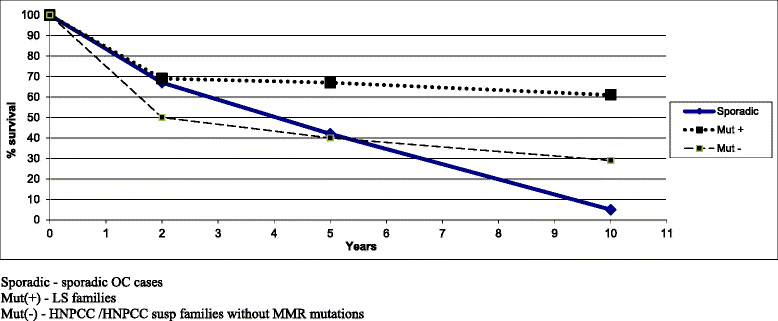
Survival comparison of the HNPCC associated ovarian cancers and unselected OC from general Polish population.

In our series of 21 OC from Polish LS families (3 OC were excluded from survival calculations due to incomplete follow up data) the 10-year survival rate was 61%. It was significantly higher when compared to 5% of 10-year survival rate of 600 consecutive patients with OC (HNPCC patients were excluded) diagnosed at our center (Figure 1). This indicates a better prognosis of Lynch syndrome-associated OC. However, 6 out of 19 (32%) affected patients from LS families died due to OC within 2 years of diagnosis. All but one patient was diagnosed after their 37th year of age (the youngest woman was 31 at the time of cancer detection). The 10-year survival rate of OC in our HNPCC subgroup was 21% (Figure 1).

## Discussion

### Management of ovarian cancer

Majority of LS-associated ovarian cancers show non-serous histology- an association between endometrioid and clear cell ovarian carcinomas and hereditary predisposition due to MMR gene mutation has been reported [30],[31]. There is limited evidence to establish whether routine screening with serum CA125 levels for the high-risk population would result in a decrease in mortality from OC. CA125, mostly associated with serous OCs, has been reported to to be elevated in around 11% of the Lynch syndrome or hereditary carcinoma of the breast/ovary cases; during the premenopausal period rising values of CA125 were associated with benign pelvic diseases,such as endometriosis, adenomyosis and myomas [32]. However, current guidelines for gynecological screening in HNPCC recommend transvaginal ultrasound (TVUs) and CA125 testing, every 1-2 years starting at 30-35 years of age [33]-[35]. Recently it has been suggested that HE4 marker might be useful for diagnosing OC due to its high specificity, especially in the premenopausal population and combining of HE4 and CA125 could be considered as an option in the OC management [36],[37].

We found four published studies that included a total of 585 women screened for the screening method included both TVUS and CA125 (applied in three studies) or TVUS only (one study). Taken together, 5 OC were diagnosed in these studies; only 1 OC during surveillance (stage IIIC), the other 4 OC were detected accidentally because of bleeding or surgery performed due to unrelated reasons and not by surveillance [38]-[41]. In all studies no benefit was shown for OC surveillance or the diagnosis of early stage ovarian cancer OC (Table 2).

**Table 2:** Literature data regarding gynecological cancer management in Lynch syndrome families

Author	Year	Patients	Methods	Outcome/drawback	References
Ketabi Z	2014	236 women from LS families/8 years	Biennial TVUs/ CA125/ES	1 OC (II B) and 3 interval OCs diagnosed	[23]
Gerritzen LH	2009	100 LS carriers or AMS members/2,5 years	TVUs/CA125/ES	1 OC diagnosed (III C)	[38]
Rijcken FE	2003	41 LS carriers or AMS members/5 years	TVUs/CA125/optional ES	1 interval EC detected	[39]
Dove-Edwin I	2002	269 AMS females/826 person-years	TVUS only	2 cases of EC- neither case detected by surveillance	[40]
Renkonen-Sinisalo L	2007	175 LS carriers/759 person-years	TVUs/CA125/routine ES	4 OC occured, none detected by surveillance	[41]
Stuckless S	2013	174 MSH2 carriers/5 years	TVUs/CA125/ES	3 deaths due to OCs both in screened and unscreened females	[42]
Schmeler KM	2006	315 LS carriers/10 years	Surgery	OC: 0/47 cases bilat salpino-oophorect. (0%) vs 12/223 no surgery (5.5%) EC: 0/61 hysterectomy (0%) vs 69/210 no surgery (33%) Surgical complication rate 1.6 percent, no data regarding overall survival	[43]
Manchandra R	2012	41 women from LS families/8 years	TVUs/routine ES	TVU alone missed 1 EC and 1 premalignant lesion	[56]

TUV – transvaginal ultrasound.

ES – endometrial sampling.

A second retrospective study reported on impact of gynecological screening in 174 MSH2 carriers [42]. Six of the 54 women in the screened group (TVUS + CA125/5 years) developed OC and there were 2 deaths due to OC within the first 2 years after diagnosis. The same number (six) of 54 matched women (taking into account age at entry into screening program) in the non-screened group developed OC, which lead to 3 deaths. In total, 16 OC among 120 unscreened women occurred, from which 5 deaths had occurred within 2 years. The authors concluded that screening did not result in any earlier cancer detection and despite screening, 2 young women died from OC. The authors suggest that prophylactic surgery be considered in female mutation carriers who have completed childbearing [42] to reduce their risk of presenting with incurable disease.

Recently an impact of gynecological screening (biennial TVUS + CA125 in case of abnormal findings/8 years) was retrospectively evaluated in 236 women (2067 women years) from Danish LS families. Four cases of OC were diagnosed: only one OC in a woman with no symptoms was detected by surveillance (highly differentiated endometrioid adenocarcinoma, stage II B); three other OCs were diagnosed as interval cancers due to symptoms [23]. Consistently with previous reports, the study showed OC screening in female LS patients to be futile.

In 2006 risk-reducing bilateral salpingo-oophorectomy has been shown to be an effective strategy for preventing OC [43]. Modeling studies of prophylactic surgery versus gynecologic surveillance for LS women showed that risk-reducing surgery is associated with the lowest costs and would increase life-expectancy [44]-[46].

According to the revised guidelines for the clinical management of Lynch syndrome prophylactic oophorectomy can be an option to be discussed with mutation carriers who have completed their families especially after the age of 40 years [11].

Given the published evidence and our own data showing a high risk of OC for young women from LS families and the absence of any positive effect of screening we conclude that it is justified to recommend the option of prophylactic oophorectomy for female carriers of MMR mutations after 35 year of age. Since three cases of primary peritoneal cancers after prophylactic bilateral salpingo-oophorectomy in HNPCC patients have been reported [47],[48] and previous studies involving women with BRCA mutations have also reported an incidence of primary peritoneal cancer after prophylactic bilateral salpingo-oophorectomy of 0.8 to 1.0 percent. [49],[50], annual TVUS and CA125 screening might be considered as an option of the follow-up after prophylactic surgery in these women. Conflicting data regarding the prognosis of HNPCC-associated OC can be found in the literature [28],[29]. But even if the results of a comparison of the 10-year survival between our LS-OC and Polish consecutive OCs pointed at a better prognosis for OC associated with MMR gene mutations, risk-reducing surgery performed at the age of 35 would save at least 6 young women from our HNPCC mutation families. Preventive oophorectomy was reported to be associated with an 80% reduction in the risk of ovarian, fallopian tube, or peritoneal cancer in BRCA1 or BRCA2 carriers and a 77% reduction in all-cause mortality [51], even if it has been observed that *BRCA1*/*2*-associated OC patients have a longer progression-free survival and overall survival compared with women with sporadic OC [52],[53]. Approximately one-third of Polish LS-associated OC patients died within two years after diagnosis, most probably due to different chemosensitivity of their tumors. Ovarian cancers are usually treated by platinium-based therapy. Contrary to serous sporadic OC and BRCA1-related OC, decreased susceptibility to cisplatin therapy has been suggested to be characteristic feature for Lynch syndrome-associated OC [54].

### Management of endometrial cancer

We found six reported studies that included a total of 1518 women screened for EC [23],[38]-[41],[55]. In the studies that used TVUS as the only screening method, interval ECs were diagnosed [40],[55]. In the studies in which protocols also included endometrial biopsies the detection of premalignant lesions and EC was significantly improved. A comparison of the results of routine endometrial biopsy [38],[41] and optional biopsy (performed in cases with abnormalities - bleeding, irregular endometrium, endometrium thickness >4 mm [23],[39] or >5 mm [38] in postmenopausal women) revealed better efficiency in disease detection in the protocols that included routine biopsies where the EC detection rate exceeded 70% in comparison to a 50% detection rate when optional sampling was performed.

Additionally, in another recent report two endometrial carcinomas and two premalignant lesions were identified in 41 women who underwent 69 annual visits with standard TVU and outpatient hysteroscopy with endometrial sampling. Screening with TVU alone would have missed one endometrial carcinoma and one premalignant lesion in that study [56].

Unlike others, Helder-Woolderink et al*.*[57] reported a lack of additional value in endometrial sampling for EC detection. 75 LS patients or their first-degree relatives at 50% risk of carrying the MMR mutation were analysed in this study, including women who underwent annual screening program based upon TVUS alone and women screened by both TVUS and routine endometrial sampling. No interval EC was diagnosed.

A large meta-analysis of sporadic endometrial cancers advocated Pipelle endometrial sampling as an equally effective, if not superior, method compared with transvaginal ultrasound for detecting endometrial cancer in both pre- and postmenopausal women [58]. A recent prospective study showed that conducting endometrial sampling at the time of colonoscopy (for colorectal cancer risk assessment) is a patient-centered option that is feasible, acceptable, and may improve adherence to LS screening recommendations [59].

Comparison of the results of the screening performed in 236 LS patients revealed that EC surveillance should only be targeted to this group of women [23].

Until now no prospective study has evaluated the impact of the EC screening on the survival of LS or HNPCC patients and the efficiency of screening for EC that generally presents with symptoms at an early stage is not clear. However, given the high risk of EC in female LS patients, according to the revised guidelines for the clinical management of LS, transvaginal ultrasound and aspiration biopsy (starting from the age of 35-40 years) should be offered as an appropriate risk reducing strategy [11]. Recently a retrospective study was published on the impact of gynecological screening in 174 MSH2 female carriers [42]. Nine of 54 women in the screened group (TVUS + endometrial sampling/5 years) developed EC, with no deaths due to this malignancy. Twenty of 54 matched women (taking into account age at entry into screening program) in the non-screened group developed EC, which resulted in 3 deaths. In total, 44 ECs among 120 unscreened women occurred, leading to 11 deaths [42]. The authors argued that risk-reducing hysterectomy and bilateral salpingo-oophorectomy after childbearing is complete is the most effective risk reduction strategy.

Modeling studies have shown that prophylactic hysterectomy and bilateral salpingo-oophorectomy can increase life expectancy and can also be a cost-effective strategy [44]-[46]. Total hysterectomy was shown to significantly reduce the risk of EC in women with Lynch syndrome [43]. However, given better outcomes than those observed for OC, the screening efficiency in detecting EC and high survival rates for this cancer [60] it remains unclear whether surgical prevention of EC in MMR carriers would significantly impact on morbidity and mortality.

In conclusion, our present knowledge on Lynch syndrome indicates that special prevention and treatment options should be applied to patients with LS. Due to the increased risk of OC, conflicting data regarding the prognosis of this disease in HNPCC and lack of any benefit from gynecological screening, it is recommended that prophylactic oophorectomy for female carriers of MMR mutations after 35 year of age should be considered as a risk reducing option. Annual transvaginal ultrasound (TVUS) supported by CA125 or HE4 marker might be considered as an option of the follow-up after prophylactic surgery in these women.

Due to the high risk of EC it is reasonable to offer, after the age of 35 years, annual clinical gynecologic examinations with transvaginal ultrasound (TVUS) supported by routine aspiration sampling of the endometrium to women from either LS or HNPCC families. Another option that could be taken into consideration for women preferring surgical prevention as a risk reducing alternative is total hysterectomy (with bilateral salpingo-oophorectomy) for carriers after childbearing is complete.

## Authors’ original submitted files for images

Below are the links to the authors’ original submitted files for images. Authors’ original file for figure 1
