# Supplementary figures and images for: Management of ovarian and endometrial cancers in women belonging to HNPCC carrier families: review of the literature and results of cancer risk assessment in Polish HNPCC families
Source: Hered Cancer Clin Pract. 2015 Jan 16;13:3. doi: 10.1186/s13053-015-0025-2 (PMC4300044; doi:10.1186/s13053-015-0025-2)

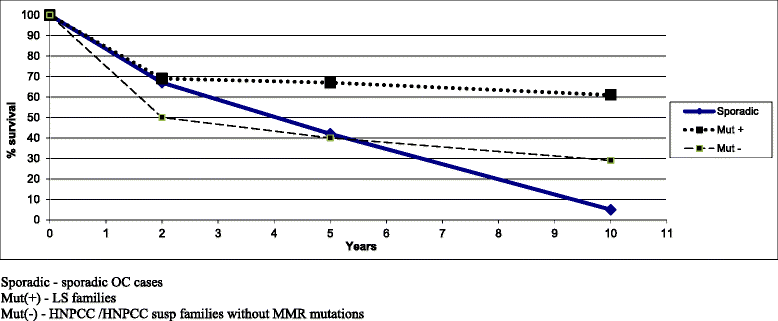

Supplement: Supplementary file 1 — Authors’ original file for figure 1 [file 13053_2015_25_MOESM1_ESM.gif]
